# Comprehensive genetic screening of the South African Parkinson's disease study collection using the NeuroBooster array

**DOI:** 10.1016/j.prdoa.2026.100508

**Published:** 2026-10-02

**Authors:** Kathryn Step, Lusanda Madula, Nicole Kuznetsov, Elizna Maasdorp, Jonathan Carr, Riaan van Coller, Marlo Möller, Ignacio F. Mata, Soraya Bardien, on behalf of the Global Parkinson's Genetics Program (GP2)

**Affiliations:** a South African Medical Research Council Centre for Tuberculosis Research (CTR); Division of Molecular Biology and Human Genetics, Faculty of Medicine and Health Sciences, Stellenbosch University, Cape Town, South Africa; b Biomedical Research Institute (BMRI), Faculty of Medicine and Health Sciences, Stellenbosch University, Cape Town, South Africa; c Center for Alzheimer's and Related Dementias (CARD), National Institute on Aging and National Institute of Neurological Disorders and Stroke, National Institutes of Health, Bethesda, MD, USA; d DataTecnica LLC, Washington, DC 20037, USA; e Division of Immunology, Faculty of Medicine and Health Sciences, Stellenbosch University, Cape Town, South Africa; f School for Data Science and Computational Thinking, Stellenbosch University, Cape Town, South Africa; g Center for Bioinformatics and Computational Biology, Stellenbosch University, Cape Town, South Africa; h Division of Neurology, Department of Medicine, Faculty of Medicine and Health Sciences, Stellenbosch University, Cape Town, South Africa; i South African Medical Research Council/Stellenbosch University Genomics of Brain Disorders Research Unit, Stellenbosch University, Cape Town, South Africa; j Department of Neurology, Faculty of Health Sciences, University of Pretoria, Pretoria, South Africa; k National Institute for Theoretical and Computational Sciences (NITheCS), Stellenbosch, South Africa; l Genomics for Health in Africa (GHA), Africa-Europe Cluster of Research Excellence (CoRE), South Africa; m Genome Sciences and Systems Biology, Cleveland Clinic Research, Cleveland Clinic Foundation, Cleveland, United States

**Keywords:** Parkinson's disease, South Africa, Pathogenic variant screening, Copy number variants, CNV-finder, SNCA, PRKN, Multiplex ligation-dependent probe amplification

## Abstract

**Introduction:**

Although there have been significant advances in the genetic etiology of Parkinson's disease (PD), most of this research has been conducted in European populations. This study aimed to characterize the genetic landscape of PD in South Africa, whose populations are renowned for their extensive genetic diversity and admixture.

**Methods:**

We recruited 689 PD probands across South Africa. These participants were genotyped using the NeuroBooster array in collaboration with the Global Parkinson's Genetics Program (GP2). An in-house computational workflow was used to identify pathogenic missense variants, which were validated using Sanger sequencing. The CNV-Finder pipeline was used to screen for copy number variations (CNVs), which were validated using Multiplex Ligation-dependent Probe Amplification (MLPA). Previously identified pathogenic variants were used as positive controls in our analysis.

**Results:**

We identified 16 unique missense variants in 47 individuals, 15 of which were independently confirmed by Sanger sequencing. However, these variants are not considered likely to be associated with PD status. We also identified new homozygous *PRKN* CNVs in three individuals and a whole-gene *SNCA* duplication in one individual, all of which were validated by MLPA. In addition, one individual had a CNV in the 5′ untranslated region of *SNCA*, although its pathogenicity remains unclear. When these findings were combined with our previous results, 3.5% of probands (24 individuals) were found to have a genetic basis for their disease.

**Conclusions:**

We conducted the largest pathogenic variant screening to date in this unique and underrepresented population, and our findings highlight the rare contribution of known pathogenic variants. This study underscores the importance of expanding genetic research in African cohorts to improve global understanding of PD etiology.

## Highlights

• Largest South African Parkinson's disease (PD) mutation screening effort to date.
• Missense and copy number variations (CNVs) identified in seven PD genes.
• *GBA1* and *PRKN* were the most frequently implicated genes.
• A novel CNV detected in the 5′ untranslated region of *SNCA.*
• Only 3.5% of probands have a genetic basis for their disease.

## 1 Introduction

Parkinson's disease (PD) is a common neurodegenerative disorder with a complex disease etiology [1]. Understanding the genetic etiology of PD can clarify the biological pathways involved in disease development, inform risk prediction, and improve strategies for disease prevention, diagnosis, and management [2].

Approximately 5–10% of individuals with PD carry a pathogenic disease variant in a single gene, representing monogenic PD [1]. In many cases, monogenic forms of the disease are attributed to highly penetrant and rare genetic variants [2]. Several genes have been linked to PD and exhibit either autosomal dominant or recessive inheritance. Notably, of these, six genes (*LRRK2*, *PRKN*, *SNCA*, *PINK1*,

*PARK7/DJ-1*, and *VPS35*) have been unequivocally linked to monogenic PD and are commonly included in PD diagnostic panels [3]. Variants in *GBA1*, although not strictly classified as monogenic, remain one of the most significant disease risk factors [4].

In addition to missense variants and small indels (short DNA insertions or deletions), the disease etiology can be attributed to genetic structural variants, resulting in a different number of DNA segment copies than expected [5]. These are known as copy number variations (CNVs) and include deletions, duplications, insertions, and genomic rearrangements. Although CNVs have been shown to play a role in PD pathogenicity [6], they are generally rare and observed in a small proportion of affected individuals at any given gene locus. Notable CNVs in the context of PD, commonly include exonic deletions and duplications in *PRKN* and *PINK1* as well as whole-gene multiplications of *SNCA* [5].

Although significant progress has been made in understanding the genetic basis of PD, most studies have focused on European populations, leaving the genetic etiology of PD in African populations largely uncharacterized. South Africa provides a particular informative setting for genetic studies because of its diverse ancestral contributions, substantial genetic variation and complex population history [7], [8]. Previous PD research in South Africa employed various wet-lab screening techniques to detect pathogenic missense variants, indels, and CNVs that are commonly observed in populations of predominantly European ancestry. However, although screening was limited to a small subset of the study collection, these studies revealed that known PD genetic causes are relatively rare in this cohort [1], [6], [9].

To address this knowledge gap, we performed a comprehensive genetic screening using genotyping array data for the South African PD Study Collection to identify pathogenic variants, including CNVs and sought to validate these findings using independent techniques (Supplementary Fig. 1).

## 2 Methods

### 2.1 Study participants and genotyping

Study participants were recruited as part of the South African PD Study Collection. PD probands (*n* = 689; Supplementary Table 1) were diagnosed using the Queen's Square Brain Bank Criteria [10], with the exclusion criterion of more than one affected relative not applied. Related affected individuals were retained to facilitate identification of shared candidate variants and assessment of potential familial variant segregation. Ethical clearance was obtained from the Health Research Ethics Committee, Stellenbosch University (2002C/059). All participants provided written informed consent and research was conducted in accordance with the Declaration of Helsinki. The participants were genotyped using the NeuroBooster array in collaboration with the Global Parkinson's Genetics Program (GP2) [11]. Previous genetic ancestry analysis has shown that individuals in the South African PD Study Collection, from which the present cohort was derived, are multiway admixed, with mean ancestry proportions of 56% European, 18.8% African, 13% indigenous Nama, 6.9% South Asian, and 5.2% Malay [12]. Local ancestry inference was performed as previously described [12].

### 2.2 Single-nucleotide variant screening file preparation and processing

This study made use of array genotyping data. For the pathogenic single-nucleotide variant (SNV) screening, quality control (QC) was performed using PLINK v1.9 [13]. Data missingness was filtered at 10% in individuals and 5% in variants, individuals with sex discrepancies were removed (female F: 0.5; male F: 0.8), variants that deviated from Hardy-Weinberg equilibrium (controls: 1 × 10^−6^; cases: 1 × 10^−10^), and those that were more than 3 standard deviations from the mean heterozygosity rate were removed.

An in-house computational workflow was developed to identify and prioritize pathogenic missense variants. Briefly, this consisted of file preparation and conversion from PLINK binary files to variant call format files. Variants were annotated using ANNOVAR v2020-06-08 with all essential databases in GRCh38. The analysis was performed in two stages. First, the computational workflow was optimized using a subset of probands (*n* = 13) with five pathogenic missense variants previously identified through previous wet-lab screening efforts (Supplementary Table 2). This ensured that the computational workflow could reliably detect known pathogenic variants. In the second stage, the analysis was restricted to individuals without a previously established genetic cause of disease (*n* = 645). The ANNOVAR output was aligned to the original files to confirm which individual carried which variant. This was followed by restricting the annotated phenotype to “*Parkinson*” or “*Parkinsonian*”. The files were examined using the following *in-silico* pathogenicity prediction tools and criteria, with variants required to meet at least one of the thresholds to be included [14]: SIFT score < 0.05, FATHMM score < 0, PolyPhen-2 score > 0.5, CADD score ≥ 20, MetaLR >0.8, and MetaSVM >0.8. Finally, variants were filtered using ClinVar (https://www.ncbi.nlm.nih.gov/clinvar/; July 2025), retaining only those classified as “pathogenic”, “likely pathogenic”, or “conflicting pathogenicity” due to their reported associations with the disease. Where DNA from family members was available, co-segregation of the pathogenic variants was assessed using Sanger sequencing. Additionally, variants of unknown significance (VUS) were screened using the same workflow, with modifications adjusting the clinical significance filtering step to include variants classified as “uncertain significance” instead of “pathogenic” or “likely pathogenic” according to ClinVar (https://www.ncbi.nlm.nih.gov/clinvar/; accessed July 2025). The prioritized VUS were not confirmed using Sanger sequencing. Additionally, all identified variants in *GBA1* were analyzed with GBA1-PD Browser v1.0 [4].

### 2.3 Validation of single-nucleotide variants

The putative pathogenic missense variants identified were confirmed using Sanger sequencing. This included primer design, polymerase chain reaction (PCR) optimization, and Sanger sequencing. The oligonucleotide primers were designed using the Integrated DNA Technologies PrimerQuest™ tool and *in-silico* PCR with the University of California Santa Cruz *in-Silico* PCR (https://genome.ucsc.edu/cgi-bin/hgPcr). Detailed information is included in Supplementary Table 3. A gradient PCR was run using the Eppendorf Mastercycler® X50 to optimize the annealing temperatures for the reactions. Following optimization, the PCR reactions were run using the Applied Biosystems GeneAMP® 2700 (Supplementary Tables 4 and 5). The PCR products were viewed using 2% agarose gel electrophoresis. Due to the presence of the highly homologous *GBA1LP* pseudogene, the aforementioned protocol was modified when validating the variants in *GBA1*. For this, genomic DNA was amplified using long-range PCR targeting a 7 kb fragment, which was viewed on a 0.5% agarose gel and exon-specific primers (nested primers) were used for Sanger sequencing. All Sanger sequencing was performed at the Central Analytical Facilities DNA Sequencing Unit, Stellenbosch University (Supplementary Methods). The DNA chromatograms were analyzed using Molecular Evolutionary Genetics Analysis v12 [15].

### 2.4 File preparation and implementation of CNV-finder for copy number variation detection

CNV-Finder was applied to the dataset using the workflow as previously described [5]. Through prior CNV screening, the South African PD Research Group had identified PD-associated CNVs in 14 probands and six family members (Supplementary Table 6). These previously validated findings were used as reference points to confirm that CNV-Finder was accurately detecting true CNVs in the current dataset. The focus of the screening for the present study was on the probands without a previously identified PD pathogenic variant, including an investigation of potential compound heterozygous CNVs when combined with the SNV analysis.

Briefly, SNV metric files were created from the genotyped IDAT files as input for the CNV pipeline using the GenoTools IDAT processing [5]. For this analysis, the GRCh38 was used as a reference genome. The input SNV metric files were split into interval-based windows to generate sample-level feature matrices for downstream analysis. We applied pretrained Long Short-term Memory models in CNV-Finder, which were trained separately for deletions and duplication, using features based on B Allele Frequency, Log R Ratio, and dosage statistics. For this, deletions were identified using a Log R Ratio of < −0.2, duplications with a Log R Ratio of >0.2, and insertions with a B Allele Frequency of 0.15–0.35 or 0.65–0.85 [5]. The analysis focused on genes/regions of interest which commonly harbor PD CNVs, including *PRKN*, *SNCA, PINK1* and *PARK7/DJ-1*. This included a default 250 kb buffer region for *PARK7/DJ-1*, *PINK1*, and *PRKN* as well as a 1000 kb buffer region for *SNCA* to adequately capture the whole-gene variations in this gene [5]. The final output of CNV-Finder includes app-compatible files for visualization using an interactive web application, and the results were visually inspected for structural variants.

### 2.5 Validation of copy number variations

The CNVs identified were confirmed using MPLA. For this, DNA was extracted from blood samples using established methods. The commercially available SALSA® MLPA® P051 Parkinson Mix-1 kit by MRC Holland (Amsterdam, The Netherlands; http://www.mlpa.com) was used for the MLPA analysis, which included probes for *PARK7/DJ-1*, *ATP13A2*, *PINK1*, *PRKN*, and *SNCA*. The analysis was conducted at the Unistel Medical Laboratories in Cape Town, South Africa, according to the manufacturer's protocol. The MLPA assays were run with positive reference samples (with known CNVs) and wild-type controls, and the results were analyzed using the Coffalyser.Net v.250317.1029 software.

## 3 Results

### 3.1 Bioinformatic analysis confirmed the presence of pathogenic and putative pathogenic missense variants

As part of developing and optimizing the pathogenic variant workflow, we confirmed that the workflow was able to detect four previously reported pathogenic variants, which were present in 11 individuals in the South African PD Study Collection. Of the 13 individuals known to carry pathogenic variants, one failed QC due to high SNP missingness, and another, despite passing QC, did not have their pathogenic variant (*PINK1* p.Y258X) genotyped. This reflects a common limitation of genotyping arrays, which often fail to capture very rare pathogenic variants.

When looking at the subset of individuals without a known genetic cause for PD, 637 successfully passed QC. After filtering for PD phenotypes and using *in-silico* pathogenicity prediction tools, 69 variants were identified across nine chromosomes. After further clinical significance filtering to retain variants classified as “pathogenic” or “likely pathogenic”, 16 unique heterozygous variants in 47 individuals were prioritized. These were in the following seven genes: *GBA1* (*n* = 18), *PRKN* (*n* = 14), *LRRK2* (*n* = 5), *PLA2G6* (*n* = 5), *PINK1* (*n* = 3), *POLG* (*n* = 1), and *SYNJ1* (*n* = 1). These 16 variants represent variants prioritized for further investigation based on their annotation and predicted clinical significance and should not be interpreted as definitive genetic causes of PD in all 47 individuals. However, it is important to note that not all of these variants are considered likely to be related to the PD status of the individuals. In particular, the heterozygous variants in *PINK1*, *PLA2G6*, *POLG*, and *SYNJ1* are in genes primarily associated with autosomal-recessive PD, while the significance of heterozygous variants in *PRKN* remains uncertain. One individual carried heterozygous variants in two different recessive PD-associated genes, *PLA2G6* (p.G361S; rs143826762) and *PRKN* (p.P437L; rs149953814). Local ancestry was incorporated into the pathogenic variant screening to determine the inferred ancestry of the genomic segment harboring each candidate variant. Among the 47 variants identified, 36 could be assigned to a single inferred local ancestry, comprising 28 European, 3 African, 3 South Asian, and 2 Nama ancestry assignments (Table 1). For the remaining 11 variants, local ancestry could not be uniquely assigned because the variant could correspond to either chromosomal strand following prior quality-control filtering. Consequently, each of these variants had two possible local-ancestry assignments. Sanger sequencing confirmed 15 of the 16 prioritized variants (Table 1; Supplementary Table 7). The variant rs191486604 (p.G430D) in *PRKN*, was not validated, suggesting a potential false-positive call in the initial workflow. The GBA1-PD Browser v1.0 classified the two *GBA1* variants as mild risk variants [4].

**Table 1:** Single nucleotide variants identified with the computational workflow which are classified as pathogenic, likely pathogenic, or conflicting pathogenicity.

GP2 sample ID/ our lab ID	Gene	Variant (Amino acid change/ rsID)	Family history of PD (additional information)	Age at onset (years)	Inferred local ancestry across genomic region encompassing the variant	Self-reported ancestry†
Dominant inheritance/ incomplete penetrance						
000163/ 10.111	*GBA1*	p.T408M / rs75548401	No	37	EUR	White
000286/ 95.41	*GBA1*	p.T408M / rs75548401	No	60	EUR	White
000339/ 96.91	*GBA1*	p.T408M / rs75548401	Yes (Brother)	72	EUR	White
000355/ 97.37	*GBA1*	p.T408M / rs75548401	No	57	EUR	White
000510/ 12.797	*GBA1*	p.T408M / rs75548401	No	79	EUR	South African Coloured
000633/ 13.509	*GBA1*	p.T408M / rs75548401	No	65	EUR	White
000066/ 67.7	*GBA1*	p.T408M / rs75548401	No	72	EUR	White
000113/ 81.61	*GBA1*	p.T408M / rs75548401	No	57	EUR	Afrikaner
000133/ 82.45	*GBA1*	p.N409S / rs76763715	No	58	EUR	White
000176/ 83.41	*GBA1*	p.N409S / rs76763715	No	42	EUR	White
000206/ 88.29	*GBA1*	p.N409S / rs76763715	No	80	SAS	South African Coloured
000357/ 97.43	*GBA1*	p.N409S / rs76763715	No	63	EUR	White
000417/ 10.327	*GBA1*	p.N409S / rs76763715	No	57	EUR	Afrikaner
000427/ 11.59	*GBA1*	p.N409S / rs76763715	No	71	EUR	Afrikaner
000580/ 13.266	*GBA1*	p.N409S / rs76763715	No	48	MAL/ EUR*	South African Coloured
000599/ 13.352	*GBA1*	p.N409S / rs76763715	No	72	EUR	Afrikaner
000273/ 12.87	*GBA1*	p.N409S / rs76763715	No	40	EUR	White
000146/ 82.91	*GBA1*	p.N409S / rs76763715	No	50	EUR	Afrikaner

Dominant inheritance						
000437/ 12.705	*LRKK2*	p.L119P / rs33995463	No	57	AFR	Black African
000195/ 99.48	*LRKK2*	p.R793M / rs35173587	No	53	NAMA	South African Coloured
000366/ 98.33	*LRKK2*	p.R793M / rs35173587	No	80	EUR	White
000154/ 83.00	*LRKK2*	p.R793M / rs35173587	No	54	EUR	White
000545/ 12.925	*LRKK2*	p.R1325Q / rs72546338	No	70	SAS	South African Coloured
000236/ 92.97	*POLG*	p.V1044A / rs150233690	No	66	SAS	South African Coloured

Recessive inheritance						
000293/ 95.66	*PINK1*	p.A339T / rs55831733	No	72	EUR	Afrikaner
000020/ 55.45	*PINK1*	p.A359T / rs76753586	No	65	AFR/ MAL*	South African Coloured
000052/ 65.68	*PINK1*	p.A494V / rs542258150	No	61	SAS/ EUR*	South African Coloured
000107/ 81.06	*PINK1*	p.A494V / rs542258150	No	58	SAS/ NAMA*	South African Coloured
000248/ 94.58	*PLA2G6*	p.S34L / rs147948449	Yes (Paternal grandmother)	62	EUR	Afrikaner
000458/ 12.061	*PLA2G6*	p.S34L / rs147948449	No	63	EUR	Afrikaner
000623/ 13.47	*PLA2G6*	p.D31N / rs150024227	No	56	EUR	Afrikaner
000050/ 65.26	*PLA2G6*	p.G361S / rs143826762	No	66	NAMA/ AFR*	South African Coloured
000074/ 11.849	*PLA2G6*	p.G361S / rs143826762	No	59	EUR	Afrikaner
000289/ 95.44	*PRKN*	p.R334C / rs199657839	No	52	EUR	White
000194/ 84.3	*PRKN*	p.R334C / rs199657839	No	50	EUR	Afrikaner
000628/ 13.497	*PRKN*	p.R334C / rs199657839	No	42	AFR/ NAMA*	Black African
000405/ 10.306	*PRKN*	p.R334C / rs199657839	No	35	EUR	Afrikaner
000552/ 12.964	*PRKN*	p.R334C / rs199657839	No	44	EUR/ AFR*	South African Coloured
000578/ 13.228	*PRKN*	p.R334C / rs199657839	No	64	AFR	Black African
000582/ 13.268	*PRKN*	p.R334C / rs199657839	No	60	NAMA/ EUR*	South African Coloured
000616/ 13.429	*PRKN*	p.R402C / rs55830907	No	37	AFR	Black African
000375/ 99.49	*PRKN*	p.R402C / rs55830907	No	62	NAMA/ EUR*	South African Coloured
000478/ 12.242	*PRKN*	p.R402C / rs55830907	No	46	EUR	White
000195/ 99.48	*PRKN*	p.R402C / rs55830907	No	67	NAMA	South African Coloured
000447/ 11.833	*PRKN*	p.P437L / rs149953814	No	52	NAMA/ AFR*	Black African
000050/ 65.26	*PRKN*	p.P437L / rs149953814	No	77	MAL/ NAMA*	South African Coloured
000060/ 12.858	*SYNJ1*	p.I707T / rs147929290	Unknown	64	EUR	Afrikaner

This table provides the variants of interest classified as “pathogenic”, “likely pathogenic”, or “conflicting pathogenicity” as of July 2025 defined by ClinVar. **†** self-reported ancestral groups labelled according to the options presented in the South African Census. * indicates variants that were excluded during quality control prior to phasing; therefore, phasing data are unavailable to determine the associated ancestral background, and both possible ancestries for the genomic region are reported. Global ancestry proportions are rounded to three decimal places. AFR, African; EUR, European; MAL, Malay; NAMA, Nama as Khoe-San proxy; SAS, South Asian; PD, Parkinson's disease.

Co-segregation analysis was performed for five families, for whom family member DNA was available (Supplementary Fig. 2) for the following four variants: rs75548401 (p.T408M) and rs76763715 (p.N409S) in *GBA1* as well as rs199657839 (p.R334C) and rs149953814 (p.P437L) in *PRKN*. Several variants were detected in unaffected relatives, precluding co-segregation with PD. This observation is consistent with *PRKN*, where carriers may be heterozygous while probands are homozygous or compound heterozygous, and for *GBA1*, which is known to exhibit incomplete penetrance. The absence of symptoms among young carriers limits definitive conclusions, underscoring the need for continued clinical follow-up. Additionally, 18 VUS were identified in 27 unrelated cases in the following genes: *SYNJ1* (*n* = 8), *PINK1* (*n* = 6), *PRKN* (*n* = 4), *GBA1* (*n* = 3), *FBXO7* (*n* = 2), *LRRK2* (*n* = 2), *HTRA2* (*n* = 1), and *PARK7/DJ-1* (*n* = 1) (Supplementary Table 8). The GBA1-PD Browser v1.0 classified the *GBA1* VUS variants as unknown severity [4].

### 3.2 Copy number variant analysis reveals pathogenic structural arrangements

The CNV analysis using CNV-Finder identified 18 exonic CNVs in *PRKN* (*n* = 15) and *SNCA* (*n* = 3) in PD probands, along with an additional *SNCA* CNV of interest in the 5′ untranslated region in one proband (Table 2 and Supplementary Table 6). The majority of the CNVs were located in *PRKN*, where ten *PRKN* CNVs were previously identified in probands and two in family members in the study collection [1], [16], [17], [18]. The new *PRKN* CNV findings include one duplication and four deletions, all of which were confirmed using MLPA, where three are homozygous. Representative examples of the graphical results from CNV-Finder are provided in Fig. 1A and B. Of the 12 exons in *PRKN*, the CNVs identified were localized to exons 2–6. Supplementary Fig. 3 provides example visualizations of a heterozygous and a homozygous *PRKN* CNV in exon 4. The three *PRKN* CNVs in the homozygous state provided a definitive genetic cause of PD, and all three individuals had no PD family history, with ages of onset ranging from 12 to 34 years (Table 2). Clinical presentations were variable, with two individuals presenting with dystonia at onset, including one initially diagnosed with dopa-responsive dystonia. One individual had a tremor-dominant phenotype, while autonomic and mild neuropsychiatric manifestations were also observed. Detailed clinical information was unavailable for one individual who was lost to follow-up. In addition, two individuals carried *PRKN* CNVs in a heterozygous state, with no second heterozygous variant identified that would support their classification as compound heterozygous carriers. Therefore, these heterozygous *PRKN* CNVs could not be considered a definitive genetic cause of PD.

**Table 2:** Newly-identified copy number variations in the present study.

GP2 ID/ our lab ID	Variant	CNV-Finder prediction value	AAO	Family history of PD	Clinical features				
					Pre-motor symptoms	Initial motor symptoms	Prominent motor features	Prominent non-motor symptoms	Treatment note
Heterozygous *PRKN* CNVs									
000478/ 12.242	Exon 2 Het DUP	1	45	No	None reported	Head and hand tremor	Freezing of gait	None	DBS for motor fluctuations
000504/ 12.729	Exon 4 Het DEL	0.9604	22	No	None reported	Dystonia	Dystonia, gait disorder	Autonomic symptoms (urge incontinence, impotence). Marked neuropsychiatric features including insomnia, depression, anxiety, and REM sleep behavior disorder. Normal cognition	DBS for motor complications

Homozygous *PRKN* CNVs									
000399/ 10.270	Exons 3–6 Hom DEL	1	21	No	Clinical details unavailable (lost to follow-up).				
000528/ 12.872	Exon 4 Hom DEL	1	34	No	None reported	Leg tremor	Tremor, off-treatment dystonia	Urge-incontinence	Levodopa induced motor complications
000648/ 13.582	Exon 2–3 Hom DEL	1	12	No	None reported	Dystonia	Abnormal eye movement, poor gait and balance with frequent falls	Mild neuropsychiatric symptoms. Normal cognition	Underwent pallidal DBS for dystonia, later revised to STN with improved outcome

*SNCA* CNVs									
000493/ 12.495	Gene Duplication	1	25	No	Muscle spasms, hyposmia, constipation, and REM sleep disturbance	Tremor	Gait and balance impairment	Dysarthria, sialorrhea, mild cognitive impairment, short term memory loss	None
000289/ 95.44	5‘UTR Duplication	1	69	Yes	Clinical details unavailable (lost to follow-up).				

The CNV-Finder prediction value represents the confidence score for each identified CNV, with values closer to 1 indicating higher confidence in the CNV call. All individuals are PD probands. AAO, age at onset; CNV, Copy number variations; DBS, deep brain stimulation; DEL, deletion; DUP, duplication; Het, Heterozygous; Hom, Homozygous; MLPA, multiplex ligation-dependent probe amplification; *PRKN*, Parkin; *SNCA*, alpha-synuclein; STN, subthalamic nuclei.

**Fig. 1:**
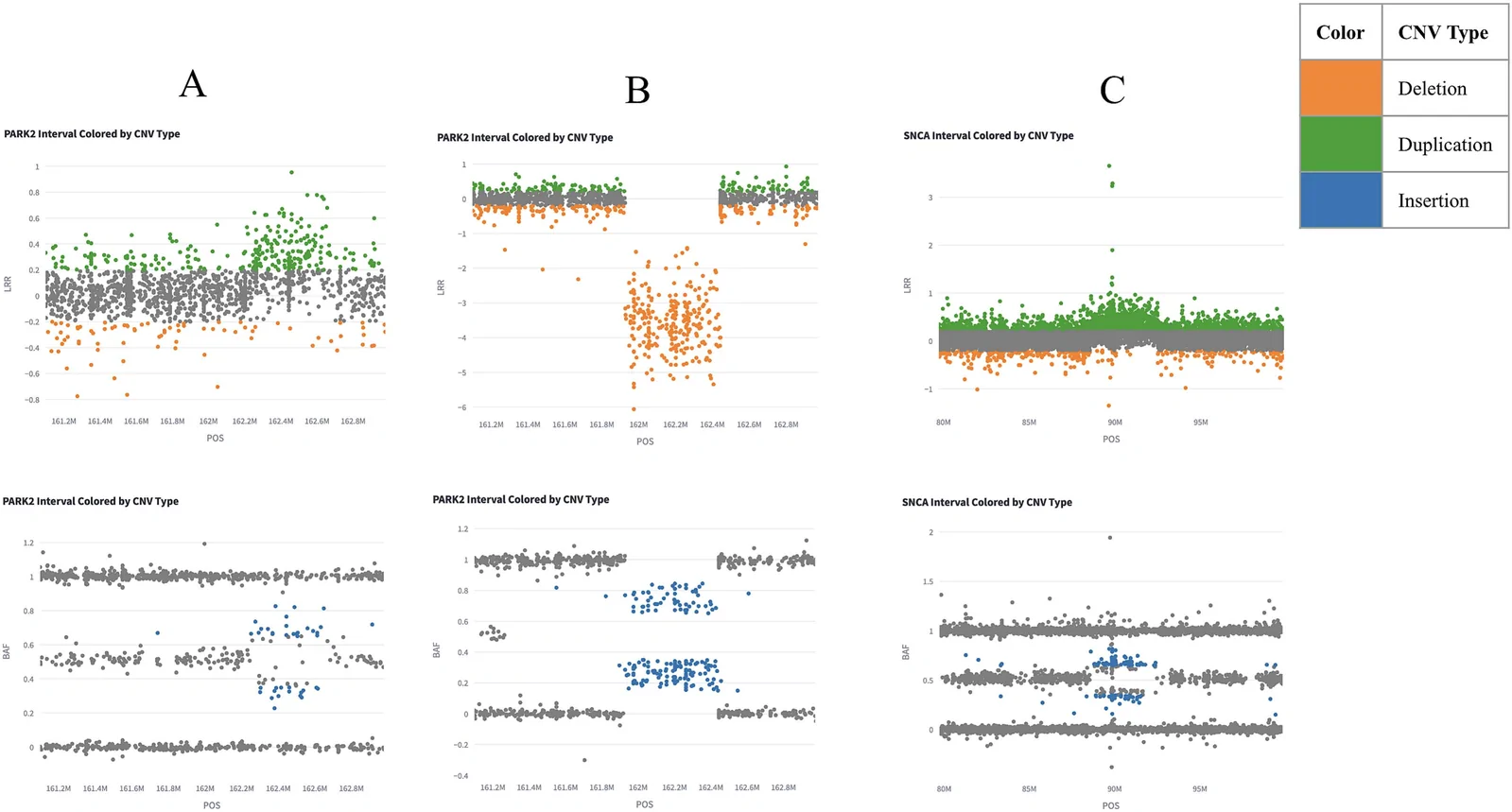
Representative CNV-Finder results showing (A) *PRKN* exon 4 duplication, (B) *PRKN* exon 4 deletion, and (C) *SNCA* whole-gene duplication. CNVs were determined using visual inspection, prediction values, and the following ranges: Deletions: Log R Ratio (LRR) < −0.2, Duplications: LRR > 0.2, Insertions: 0.15 < B Allele Frequency (BAF) < 0.35 or 0.65 < BAF < 0.85.

For *SNCA*, five whole-gene multiplications that were previously identified in two families were all confirmed with CNV-Finder. This included three individuals in one family with a gene duplication and two individuals in another family with a gene triplication [16], [19]. Moreover, one new *SNCA* whole-gene duplication was identified using CNV-Finder (Fig. 1C) and validated with MLPA. This individual has an age at onset of 25 years and no family history of PD. Clinical manifestations included resting tremor, impaired balance and coordination, and non-motor symptoms including hyposmia, sleep disturbances, constipation, and short-term memory impairment (Table 2). Interestingly, a CNV was identified in the 5′ untranslated region of *SNCA* in one case with an age at onset of 69 and a family history of PD (Supplementary Fig. 4). However, due to the intronic location of this CNV, confirmation with the MLPA P051 kit was not possible.

Detailed clinical information for each newly identified case with CNV is presented in Table 2. We also observed *PRKN* cases with two CNVs that might indicate biallelic variation. These potential pairs were already known from earlier screening efforts, but the second CNV in each case fell below the CNV-Finder prediction score threshold and was excluded, as indicated in Supplementary Table 6. When considering both the pathogenic SNV and CNV analyses, there were no new compound heterozygous variants detected in the genes associated with recessive forms of PD in the South African PD Study Collection.

Overall, the genetic findings identified in this study were considered at the subject level to distinguish variants that provide a definitive genetic cause of PD from those that were identified through variant prioritization but do not currently provide a definitive explanation for PD. The final number of individuals with a definitive pathogenic cause of PD, incorporating the findings from the current and previous screening efforts, is summarized in Table 3.

**Table 3:** Overview of the variants identified in study collection probands.

Gene	Proband carriers, n (%)	Family history of PD, n (%)	EOPD, n (%)	Probands explained, n (%)
*PARK7/DJ-1*	1 (0.15)	0 (0)	1 (0.15)	0 (0)
*GBA1*	22 (3.19)	6 (0.87)	7 (1.02)	0 (0)
*LRRK2*	14 (2.03)	6 (0.87)	3 (0.44)	9 (1.31)
*PRKN*	34 (4.93)	13 (1.89)	19 (2.76)	11 (1.6)
*PINK1*	5 (0.73)	1 (0.15)	1 (0.15)	1 (0.15)
*PLA2G6*	5 (0.73)	1 (0.15)	0 (0)	0 (0)
*POLG*	1 (0.15)	0 (0)	0 (0)	0 (0)
*SYNJ1*	1 (0.15)	1 (0.15)	0 (0)	0 (0)
*SNCA*	4 (0.58)	3 (0.44)	3 (0.44)	3 (0.44)

This table provides a summary of all genetic variants associated with Parkinson's disease identified in the South African PD study collection to date, comprising 689 probands. ‘Proband carriers, n (%)’ denotes the percentage of probands carrying a pathogenic, likely pathogenic, or variant of uncertain significance (VUS), combining results presented in this manuscript with findings from previous screening initiatives. Percentages for family history and EOPD correspond specifically to this group of proband carriers. ‘Probands explained, n (%)’ refers to the proportion of probands for whom a definitive genetic cause of PD has been established and includes only confirmed pathogenic or likely pathogenic variants. This category excludes VUS, as well as incomplete compound heterozygous findings and variants with unknown penetrance.

## 4 Discussion

This study extends our understanding of the genetic architecture of PD in South Africa, a setting characterized by substantial genetic diversity and admixture but limited representation in genomic research. When the present study's findings were integrated with previous screening of the South African PD Study Collection, only 3.5% of probands have confirmed variants that could adequately explain their disease (Table 3). This ‘diagnostic yield’ is lower than that reported in several large international PD cohorts including ROPAD and PDGENE [9], [20], [21].

The detection of potentially relevant variants in several established PD-associated genes supports the genetic heterogeneity of PD in this cohort. Nevertheless, the difference between the number of prioritized variants and the number considered definitively causal illustrates the challenges of interpreting rare variation. This is particularly important in African and admixed populations, which remain underrepresented in reference databases and variant-classification resources. Consequently, variants may remain classified as variants of uncertain significance because of insufficient ancestry-matched frequency data, functional evidence, or clinical information rather than an absence of biological relevance.

Furthermore, our findings highlight both previously reported and novel CNVs within our study collection. The identification of homozygous *PRKN* CNVs in three probands, together with their early-onset presentations, provides strong evidence that these biallelic events are the genetic cause of disease, consistent with previous reports [1]. The genomic distribution of *PRKN* CNVs in our dataset, with most occurring between exons 2 and 6 and exon 4 being the most frequently affected region, is also consistent with previous observations [6]. In contrast, two individuals carried single *PRKN* CNVs without an accompanying pathogenic SNV or second CNV. Although single *PRKN* CNVs have been proposed to increase PD risk [22], this association remains inconclusive [23], and more complex structural variants may remain undetected by genotyping array data. Long-read sequencing may therefore be useful for resolving unexplained single-heterozygous *PRKN* cases [24]. For *SNCA*, the identification of both known and novel CNVs further supports the contribution of structural variation to *SNCA*-associated PD. In particular, the *SNCA* duplication in an individual with early-onset PD is consistent with previous reports of duplications occurring in both familial and sporadic disease [[19], [23]], while the CNV identified in the 5′ untranslated region may have functional consequences through disruption of regulatory elements and transcription factor binding [23]. However, these findings should be interpreted in light of the limitations of array-based CNV calling, and independent validation using approaches such as whole-genome sequencing would be required to confirm CNVs that could not be independently validated.

The prevalence of PD-associated CNVs in individuals of African ancestry, particularly from sub-Saharan Africa, remains limited, although it is unclear whether this reflects small sample sizes, true rarity, or both. Consistent with this uncertainty, previous screening efforts identified only five probands with *PRKN* CNVs and no *SNCA* CNVs reported in individuals of African ancestry [6]. Similarly, a recent study successfully identified *PRKN* CNVs in individuals of African and African admixed ancestry on a global scale, but with no *SNCA* CNVs detected [25]. However, the reported frequency of *PRKN* CNVs in that study was 0.7% of cases, highlighting the low frequency of CNVs in African populations compared to other ancestral groups, where frequencies as high as 10.8% have been reported [26]. Taken together, this available literature suggests low CNV frequencies in African populations and suggests that novel genetic contributors remain to be uncovered.

While several variants were prioritized in the analysis, the study has limitations. Firstly, the ancestral composition of our cohort is not representative of the broader South African population [27]. Several factors may contribute to this imbalance, including convenience sampling, differences in life expectancy, unequal access to healthcare, and cultural perceptions of PD. Ongoing recruitment efforts are aimed at increasing the representation of underrepresented ancestral groups and thereby improving the generalizability of our findings to the South African population. Also, while genotyping arrays are widely used for their cost-effectiveness and coverage of common variants; they have limited sensitivity for rare variant detection and do not capture novel pathogenic variants [28]. Additionally, probe sensitivity varies across genotyping arrays, which can lead to the detection of false positives and negatives, highlighting the importance of further validation. Moreover, the detection of true CNVs using genotyping arrays remains challenging. While various bioinformatics approaches are available for this analysis, there is considerable variability in these methods [5]. Some compound heterozygous CNVs, particularly in *PRKN*, were missed because one of the CNVs in the pair received a low CNV-Finder prediction value and was therefore excluded. Furthermore, potential compound heterozygous CNVs occurring in trans across consecutive exons may also be missed without long-read sequencing or parental testing to resolve allele phase. This indicates a limitation of this tool currently and requires further refinement. The results from CNV detection must therefore be carefully evaluated, either by integrating multiple tools or through wet-lab confirmation. While this is possible for genes that are on MLPA kits, not all CNVs can be validated with this approach. Ultimately, long-read whole-genome sequencing (WGS) or array comparative genomic hybridization is required to confirm novel CNVs and complex structural arrangements, such as inversions, in this population. This includes variants in non-coding regulatory regions, such as the 5′ untranslated region of *SNCA*, which cannot be validated using MLPA, where preliminary evidence suggests this CNV may represent a true finding.

In conclusion, this study is the first large-scale SNV and CNV screening in Southern Africa and provides important insights into the genetic architecture of PD in the South African population. Our findings demonstrate the utility of genotyping array data for large-scale genetic screening, particularly in underrepresented populations where resources may be limited, while also highlighting the limitations of array-based approaches for detecting rare pathogenic variants. The availability of WGS data for our study collection will enable more comprehensive future investigation of pathogenic variants that may have been missed in the current analyses. Overall, this study provides an important foundation for future studies and highlights the need to include African populations in neurological disease research to advance a global understanding of disease etiology.

## CRediT authorship contribution statement

**Kathryn Step:** Writing – original draft, Visualization, Methodology, Investigation, Formal analysis, Data curation, Conceptualization. **Lusanda Madula:** Writing – original draft, Validation, Methodology, Investigation, Formal analysis, Data curation. **Nicole Kuznetsov:** Writing – review & editing, Visualization, Software, Methodology, Investigation, Formal analysis, Data curation. **Elizna Maasdorp:** Writing – review & editing, Supervision, Project administration, Methodology. **Jonathan Carr:** Writing – review & editing, Resources, Data curation. **Riaan van Coller:** Writing – review & editing, Resources, Data curation. **Marlo Möller:** Writing – review & editing, Supervision, Project administration, Methodology. **Ignacio F. Mata:** Writing – review & editing, Supervision, Project administration, Methodology, Funding acquisition, Conceptualization. **Soraya Bardien:** Writing – review & editing, Supervision, Resources, Project administration, Funding acquisition, Conceptualization.

## Declaration of competing interest

The authors declare the following financial interests/personal relationships which may be considered as potential competing interests: Soraya Bardien reports financial support was provided by National Research Foundation of South Africa. Soraya Bardien reports financial support was provided by South African Medical Research Council. Soraya Bardien reports financial support was provided by Centre for Tuberculosis Research (CTR) of the South African Medical Research Council. Ignacio F. Mata reports a relationship with National Institutes of Health that includes: funding grants. Ignacio F. Mata reports a relationship with The Michael J. Fox Foundation and the Aligning Science Across Parkinson’s Global Parkinson Genetic Program (ASAP-GP2) that includes: consulting or advisory and funding grants. Ignacio F. Mata reports a relationship with American Parkinson’s Disease Association (APDA) and Department of Veterans Affairs that includes: funding grants. Ignacio F. Mata reports a relationship with Parkinson’s Foundation that includes: consulting or advisory. Riaan van Coller reports a relationship with Medtronic Africa Pty Ltd that includes: consulting or advisory. Given the corresponding author’s (Soraya Bardien) and one of the co-author’s (Igacio Mata) roles as Editorial Board members of Parkinsonism & Related Disorders, they had no involvement in the peer review of this article and had no access to information regarding its peer review. Full responsibility for the editorial process for this article was delegated to another journal editor. If there are other authors, they declare that they have no known competing financial interests or personal relationships that could have appeared to influence the work reported in this paper.

## Appendix A Supplementary data

Supplementary material: Supplementary Methods, Figures, and Tables providing additional methodological details and results of genetic variant and copy number variation analyses in the South African Parkinson's Disease Study Collection.
